# Marine Drug Discovery through Computer-Aided Approaches

**DOI:** 10.3390/md21080452

**Published:** 2023-08-17

**Authors:** Susana P. Gaudêncio, Florbela Pereira

**Affiliations:** 1 Associate Laboratory i4HB, Institute for Health and Bioeconomy, NOVA Faculty of Sciences and Technology, NOVA University of Lisbon, 2819-516 Lisbon, Portugal; 2 UCIBIO, Applied Molecular Biosciences Unit, Chemistry Department, NOVA Faculty of Sciences and Technology, NOVA University of Lisbon, 2819-516 Lisbon, Portugal; 3 LAQV, Chemistry Department, NOVA Faculty of Sciences and Technology, NOVA University of Lisbon, 2819-516 Lisbon, Portugal; florbela.pereira@fct.unl.pt

## 1. Blue Biotechnology Framework

Besides the importance of our oceans as oxygen factories, food providers, shipping pathways, and tourism enablers, oceans hide an unprecedented wealth of opportunities [1]. Marine organisms and microorganisms are valuable sources of primary and secondary metabolites, biopolymers, and enzymes, which can be used as lead agents for drug discovery, filling in the pharmaceutical industry pipeline and improving their development processes (e.g., drug discovery, drug repurposing, absorption, distribution, metabolism, elimination, and toxicity (ADMET) prediction, drug delivery, among others), especially when applying computer-aided tools and methods, and also as a source of bio-inspired material for numerous medical and biotechnological applications. The field of computer-aided ligand- and structure-based methodologies for marine drug lead discovery is still developing. By assisting in the structure elucidation of secondary metabolites, repurposing known marine natural products (MNPs) for new therapeutic purposes, and identifying novel hits or leads against selected therapeutic targets, computational approaches and chemistry simulation methods can be successfully used in the discovery, design, and development of new chemical agents for therapeutic applications [2,3,4]. The eminent marine (blue) biotechnology field has gained visibility worldwide in many complementary scientific fields, inspiring the creation of several legislative, infrastructural, and scientific collaborative networks [5,6]. With computer-aided approaches playing a crucial role in advancing this scientific field, the computational tools could ultimately become a significant driver in economic development, the formation of innovative biotechnological applications, and in the accomplishment of sustainable drug discovery approaches worldwide.

## 2. Objective of Marine Drug Discovery through Computer-Aided Approaches Special Issue

The Special Issue “Marine Drug Discovery through Computer-Aided Approaches” was created with the objective of mapping the current scientific actors in the field of computer-aided approaches applied to blue biotechnology, and of providing a comprehensive overview of the great variety of advanced computer-aided methods for the discovery and identification of molecular agents with added value and health-promoting properties for the development of medical and biotechnological applications. This Special Issue invited the blue biotechnology community working with computer-aided technology to submit original research, reviews, and perspectives in all steps of the marine biotechnology development pipeline including computer-aided methods; from blue biotechnology, drug discovery, drug repurposing, chemoinformatics, bioinformatics, dereplication, MNPs databases, machine learning techniques, biological and chemical space, Quantitative Structure–Activity Relationship (QSAR), molecular docking, Computer-Aided Drug Design (CADD), and Computer-Assisted Structure Elucidation (CASE), generating a compilation of processes and technologies. The aim was also to develop a “guidebook” for maximizing the impact of marine biotechnology development that can be used to start, improve, and facilitate collaborations between related and complementary scientific fields, by providing information, expert contacts, and their expertise that will, both directly and indirectly, improve the discovery and innovation in blue biotechnology and boosting blue bioeconomy using computing methodologies. 

## 3. Topics of the Participating Research Community 

The Special Issue “Marine Drug Discovery through Computer-Aided Approaches” comprises nine articles reporting original research. These range from using computer software, machine learning, molecular docking, in silico modelling and animal modelling for dereplication, aiding MNPs structure elucidation, and the prediction of MNPs bioactivities and protein binding targets. This Special Issue is a must read for those who want to start using computer methods in marine biotechnology research. The nine contributions are described below by publication date. 

### 3.1. Predicting Antifouling Activity and Acetylcholinesterase Inhibition of Marine-Derived Compounds Using a Computer-Aided Drug Design Approach 

A CADD strategy combining ligand- and structure-based approaches was used for predicting the antifouling properties of MNPs. The QSAR classification model was constructed using antifouling screening data from 141 organic compounds that were taken from the ChEMBL database and the literature using the CADD ligand-based technique, attaining a highest prediction accuracy score of up to 71%. The best QSAR model created was also used to conduct a virtual screening campaign on 14,492 MNPs from Encinar’s website and 14 MNPs that are currently in the clinical pipeline. The 125 MNPs chosen by the QSAR approach were employed in molecular docking tests against the acetylcholinesterase enzyme in the CADD structure-based approach. The most promising marine drug-like leads as antifouling agents were identified as 16 MNPs, including macrocyclic lactam, macrocyclic alkaloids, indole, and pyridine derivatives [7].

### 3.2. Uncovering the Bioactive Potential of a Cyanobacterial Natural Products Library Aided by Untargeted Metabolomics

Numerous cyanobacteria are kept in the Blue Biotechnology and Ecotoxicology Culture Collection (LEGE-CC), but little is known about their chemical diversity. A library of natural compounds was created to speed up its bioactivity screening. Sixty strains were examined for their cytotoxic potential against 2D and 3D models of human colon cancer (HCT 116) and the non-cancerous cell line hCMEC/D3. Their metabolome was analyzed and annotated using MolNetEnhancer and processed with MetaboAnalyst, allowing the selection of seven out of sixty cyanobacterial strains for the discovery of anticancer drug leads while dereplicating the chemical content of these cyanobacteria [8].

### 3.3. Application of Networking Approaches to Assess the Chemical Diversity, Biogeography, and Pharmaceutical Potential of Verongiida Natural Products

A review of all isolated natural products (NPs) identified in the sponge’s order Verongiida from 1960 to May 2020 was performed compiling detailed information on their physico-geographical characteristics. Pharmacokinetic characteristics and possible medicinal potential of NPs from Verongiida were inferred using physico-chemical data. To comprehensively study the chemical space interactions between taxonomy, secondary metabolites, and drug score variables, a network analysis was used for the NPs made by Verongiida sponges, allowing the detection of differences and correlations within a dataset. Bipartite connection networks and scaffold networks provided a platform for investigating chemical diversity, and chemical similarity networks linking pharmacokinetic features with structural similarities, which can be used for other sponge orders or families [9].

### 3.4. Investigation of Marine-Derived Natural Products as Raf Kinase Inhibitory Protein (RKIP)-Binding Ligands

Numerous illnesses, including cancer, Alzheimer’s, and diabetic nephropathy, are associated with the aberrant expression of RKIP. RKIP also functions as a tumor suppressor, making it a desirable therapeutic target. Only a few small molecules have been identified to alter the activity of RKIP. A pharmacophore model was created to investigate the characteristics of locostatin, the most effective RKIP modulator. A MNPs library was then obtained after the generated model was put through a screening process. The in silico hits may serve as strong RKIP modulators and disrupt interactions with RKIP’s binding proteins [10]. 

### 3.5. Saliniquinone Derivatives, Saliniquinones G−I and Heraclemycin E, from the Marine Animal-Derived Nocardiopsis aegyptia HDN19-252

The Antarctic marine-derived actinomycete *Nocardiopsis aegyptia* HDN19-252 was used as a resource to produce four novel anthraquinone derivatives, including saliniquinones G–I (**1**–**3**) and heraclemycin E (**4**). Extensive NMR, MS, and ECD investigations revealed their structures, including absolute configurations. Saliniquinones **1** and **2** demonstrated encouraging inhibitory action against six tested bacterial strains, including methicillin-resistant coagulase-negative staphylococci (MRCNS), with MIC values ranging from 3.1 to 12.5 μM [11].

### 3.6. Efficacy of Chondroprotective Food Supplements Based on Collagen Hydrolysate and Compounds Isolated from Marine Organisms

The prevalence of osteoarthritis is higher in older individuals and is one of the most prevalent joint diseases in both humans and animals. The bioactivities of collagen hydrolysates, sulfated glucosamine, and specific fatty-acid-enriched dog rations were examined as prospective therapeutic options for early osteoarthritis using 52 dogs. The possibility that these well-characterized compounds may function as efficient nutraceuticals is supported by biophysical, biochemical, cell biology, and molecular modeling techniques. Animal model and molecular modeling for the receptor proteins MMP-3, TIMP-1 and ADAMTS-5 of intermolecular interactions strongly validated the applied collagen hydrolysates as well as sulfated glucosamine compounds from marine organisms. Molecular modeling simulations were employed to further evaluate the contact efficacy of collagen fragments and glucosamines with protein receptor architectures. There are potential advantages of using lipids, particularly eicosapentaenoic acid (extracted from fish oil), sulfated glycans (such as sulfated glucosamine from crabs and mussels), and collagen hydrolysates on biochemical and physiological processes for applications in dietary supplements for human and veterinary medicine [12].

### 3.7. Solid-Phase Extraction Embedded Dialysis (SPEED), an Innovative Procedure for the Investigation of Microbial Specialized Metabolites

In situ physical separation of the mycelium of filament-forming microorganisms, such as actinomycetes and fungi, and the XAD-16 resin used to trap the secreted specialized metabolites was accomplished using the novel solid-phase extraction embedded dialysis (SPEED) technology. SPEED is made up of an internal dialysis tube holding XAD resin and an exterior nylon cloth. The dialysis barrier chooses the molecular weight of the trapped chemicals and stops biomass or macromolecules from accumulating on the XAD beads. SPEED is a cultivation procedure assisted by a microbial biofilm since the external nylon encourages its creation. Marine *Streptomyces albidoflavus* 19-S21, isolated from a core of a submerged Kopara sampled at 20 m from a saltwater pond border, was subjected to SPEED technology. Using dereplication techniques based on molecular networking and thorough chemical analysis, the chemical space of this strain was successfully studied, demonstrating the influence of the culture support on the molecular profile of the secondary metabolites produced by *Streptomyces albidoflavus* 19-S21 [13].

### 3.8. Potency- and Selectivity-Enhancing Mutations of Conotoxins for Nicotinic Acetylcholine Receptors Can Be Predicted Using Accurate Free-Energy Calculations

Nicotinic acetylcholine receptor (nAChR) subtypes are important therapeutic targets, however, because of their striking similarity in sequence identities, it is difficult to pharmacologically distinguish between them. Additionally, nAChR problems may be successfully treated by using -conotoxins (-CTXs), which are naturally occurring selective and competitive antagonists for nAChRs. The primary goal of most -CTX mutagenesis investigations is to identify selectivity-enhancing mutations, although doing so with conventional docking techniques is challenging due to the lack of crystal structures for -CTX and nAChR. This study anticipates the structures of -CTXs bound to the nAChR subtypes 3 and 4 using homology modeling and re-predicts the relative potency and selectivity of -CTX mutants at these subtypes using free-energy perturbation (FEP). First, we employ the three crystal structures of the acetylcholine-binding protein, a homologue of the nAChR. The relative affinities of twenty point mutations made to the -CTXs LvIA, LsIA, and GIC using three crystal structures of the nAChR homologue, acetylcholine-binding protein (AChBP) was re-predicted, with an overall root mean square error (RMSE) of 1.08 ± 0.15 kcal/mol and an R^2^ of 0.62, equivalent to experimental uncertainty. Then, with an overall RMSE of 0.85 ± 0.08 kcal/mol and an R^2^ of 0.49, we employ AChBP as a template for 32 and 34 nAChR homology models linked to the -CTX LvIA and re-predict the potencies of eleven point mutations at both subtypes. The commonly used molecular mechanics–generalized born/surface area (MM-GB/SA) approach, which yields an RMSE of 1.96 ± 0.24 kcal/mol and an R^2^ of 0.06 on the identical data, is substantially worse than this. Moreover, in contrast to MM-GB/SA, FEP correctly categorizes 32 nAChR selective LvIA mutants. FEP was used to undertake a thorough scan for amino acid alterations in LvIA. Fifty-two of these mutations will have greater than 100X selectivity for the 32 nAChR. FEP is ideally adapted to properly forecast mutations that will increase the potency and selectivity of -CTXs for nAChRs and to find alternative methods for discovering selective α-CTXs drugs [14].

### 3.9. In Vitro and In Silico Characterization of G-Protein Coupled Receptor (GPCR) Targets of Phlorofucofuroeckol-A and Dieckol

Polyphenolic substances called phlorotannins are obtained from marine algae, particularly brown algae. Dieckol and phlorofucofuroeckol-A (PFF-A) are the two main phlorotannins among many others, and although possessing a greater range of biological activities, less is known about the G protein-coupled receptors (GPCRs) that these phlorotannins target. Twenty major protein targets were predicted by in silico proteocheminformatics modeling, and in vitro functional assays demonstrated that two phlorotannins’ primary GPCR targets had good agonist and antagonist effects at the 2C adrenergic receptor (2CAR), adenosine 2A receptor (A2AR), glucagon-like peptide-1 receptor (GLP-1R), and 5-hydroxytryptamine 1A receptor (5-TH1AR). Additionally, PFF-A had a promising agonist action at the cannabinoid 1 receptor and an antagonist effect at the vasopressin 1A receptor (V1AR) while dieckol demonstrated an antagonist effect at the V1AR. In silico molecular docking simulation enables the analysis and pinpointing of specific binding characteristics of these phlorotannins to the target proteins. According to the docking data, dieckol and PFF-A bind to the proteins’ crystal structures with good affinity and important interplaying amino acid residues equivalent to reference ligands. The primary receptors for dieckol and PFF-A are the 2CAR, A2AR, -OPR, GLP-1R, 5-TH1AR, CB1R, and V1AR [15].
